# Primary Progressive Aphasia With Parkinsonism

**DOI:** 10.1002/mds.25341

**Published:** 2013-02-11

**Authors:** Karen M Doherty, Jonathan D Rohrer, Andrew J Lees, Janice L Holton, Jason Warren

**Affiliations:** 1 Reta Lila Weston Institute of Neurological Studies, UCL Institute of NeurologyLondon, UK; 2 Dementia Research Center, UCL Institute of NeurologyLondon, UK; 3 Queen Square Brain Bank for Neurological Disorders, UCL Institute of NeurologyLondon, UK

**Keywords:** aphasia, parkinsonism, Lewy body, Alzheimer's disease, Parkinson's disease

## Abstract

A 65-year-old man presented with word-finding difficulty and gait disturbance. His speech was nonfluent with word retrieval impairment and difficulties with sentence repetition. Other cognitive domains were intact initially. He developed asymmetrical bradykinesia, rigidity and a rest tremor. Over the following 8 years, his speech production impairment slowly deteriorated with the development of a motor speech disorder, anomia, impaired repetition of single words as well as sentences, and impaired comprehension of initially sentences then single words. His parkinsonian syndrome also deteriorated with limited response to levodopa. Serial brain MRI revealed progressive asymmetric perisylvian atrophy. He died after a disease duration of 12 years. The clinical syndrome is discussed by an expert, the pathology is described, and important clinical points from the case are highlighted. © 2013 *Movement* Disorder Society

## Clinical History

A 65-year-old right-handed retired engineer (awarded a First in physics, Cambridge University, UK) presented to a tertiary referral cognitive disorders clinic with a 3-year history of word-finding difficulty and less-fluent speech. However, he did not complain of problems in nonlinguistic cognitive domains and was independent in all activities of daily living. His wife reported that he had begun to shuffle when he walked, although the patient did not himself complain of this. There was no family history of speech disorders or other neurological illness. He lost 1 point on the Mini–Mental State Examination (MMSE) because he was unable to repeat the phrase “No ifs, ands, or buts.” His general neurological examination was unremarkable at this time. When assessed neuropsychologically (Table 1), he scored at an average level on a test of naming with some phonemic errors; repetition of single words was normal, but repetition of sentences was impaired. Performance in other cognitive domains was within normal limits. Repeat neurological examination 6 months later revealed mild cogwheeling and bradykinesia of the right hand with an asymmetrical postural tremor of the right hand.

**TABLE 1:** Neuropsychometry at 3.5, 7.3, and 8.4 years from symptom onset

Disease duration, years	3.5	7.3	8.4
MMSE (/30)	29	25	23
FAB (/18)	NT	14	13
Verbal IQ	116	NT	NT
Performance IQ	137	NT	NT
Memory			
RMT Words (/50)	47	NT	NT
RMT Faces (/50)	41	NT	NT
Camden Pictorial Memory (/30)	NT	29	30
Visuoperceptual skills			
VOSP Object Decision (/20)	19	18	18
Executive function			
Trail Making Test B (scaled score)	10.0	7.6	6.4
Naming			
Graded Naming Test (/30)	19	3	1
Noun naming (/20)	NT	19	18
Verb naming (/20)	NT	17	14
Repetition			
Single word (high frequency) (/30)	30	30	23
Single word (low frequency) (/30)	30	26	17
Nonword (/20)	NT	19	15
Sentence (/20)	16	7	7
Single-word comprehension			
Synonyms: concrete words (/25)	NT	21	20
Synonyms: abstract words (/25)	NT	18	15
Sentence comprehension			
Modified PALPA 55 (/24)	NT	20	14
Verb tense comprehension (/20)	NT	20	17
Reading and spelling			
Schonell Reading Test (/100)	NT	72	60
Snowling Nonword Reading Test (/20)	NT	17	15
Baxter Graded Spelling Test (/30)	NT	13	6
Digit Span			
Forward	NT	4	5
Backward	NT	3	2

Abbreviations: FAB, Frontal Assessment Battery; RMT, Recognition Memory Test; VOSP, Visual Object and Space Perception Battery; PALPA, Psycholinguistic Assessment of Language Processing in Aphasia; NT, not tested.

At 4.8 years from symptom onset, he complained that his speech problems had worsened, with increased difficulties in holding a conversation. There were no complaints in other cognitive domains and no behavioral symptoms. Speech production remained the main cognitive feature, with a motor speech disorder, phonemic errors, and impaired speech repetition mainly affecting phrases and sentences (Table 1). There was no orofacial or limb apraxia. Motor examination then revealed a rest tremor in the right hand as well as the postural tremor, cogwheeling, and rigidity. Myoclonus was evident in both hands.

**FIG. 1:**
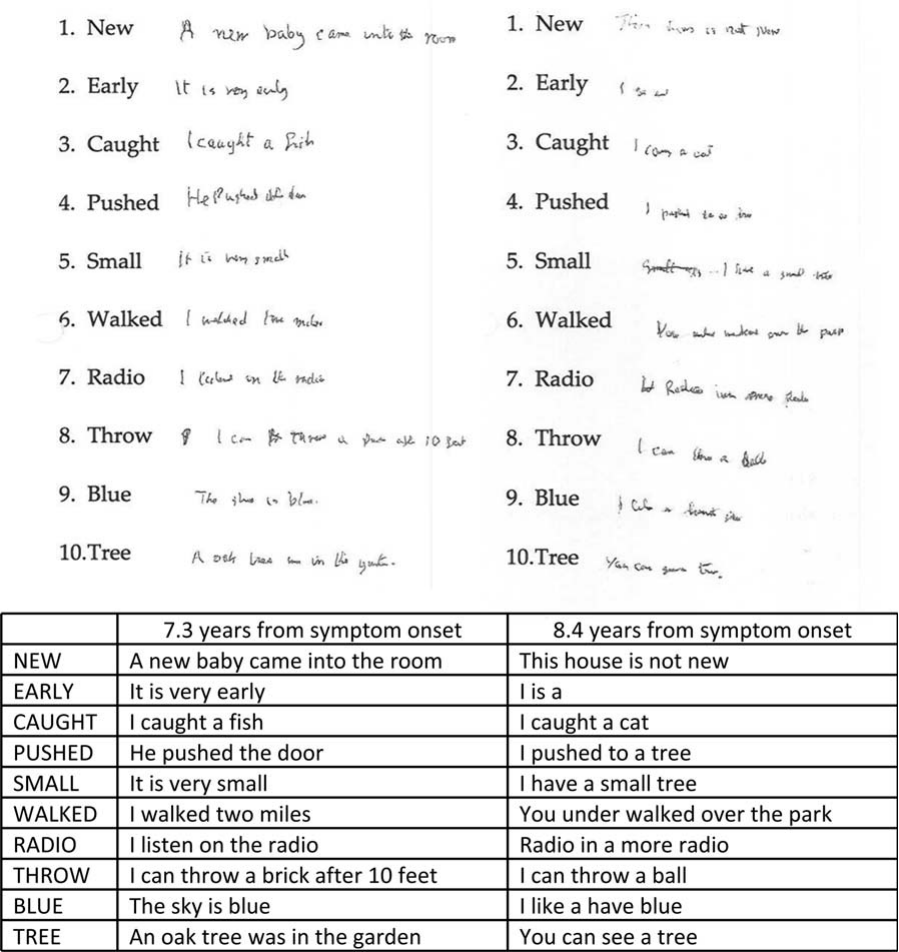
Writing task (sentence construction around the nominated word) at 7.3 and 8.4 years from symptom onset showing progressive micrographia and agrammatism.

At a further review 7.3 years from symptom onset, both language and motor aspects had deteriorated. Grammatical errors were evident when the patient expressed himself in writing (Fig. 1). There was no orofacial apraxia, but he had developed limb apraxia, predominantly affecting the right hand. He also had evidence of impaired graphaesthesia in the right hand. He had become anomic with impaired sentence comprehension and evidence of executive dysfunction on neuropsychometry (Table 1); nonlinguistic domains remained relatively intact. Parkinsonian signs were much more evident with micrographia (Fig. 1), asymmetrical bradykinesia, rigidity, and rest tremor, as well as a shuffling festinant gait with decreased arm swing on the right; eye movements were normal. Commencement of levodopa therapy (maximum daily dose achieved: 500 mg) produced an improvement in his tremor, but not the other parkinsonian features.

When next assessed 8.4 years from symptom onset, the patient's speech had become dysarthric and stuttering, with frequent phonetic and phonemic errors and increasingly severe expressive agrammatism (Fig.1). He had worsening limb apraxia and had developed orofacial apraxia. Neuropsychometry (Table 1) revealed profound anomia with more-severe impairments of speech repetition (both sentences and single words) and executive function. Sentence comprehension had deteriorated and there was evidence of impaired single-word comprehension. His performance on episodic memory and visuoperceptual tasks remained normal. When last reviewed 9.5 years from symptom onset, the patient had little intelligible speech, with a severe motor speech disorder, dysarthria, and impaired comprehension, with a moderate to severe asymmetrical rest tremor of the upper limbs, bradykinesia, and rigidity of the right upper limb. Therapy with l-dopa was felt to have produced only limited benefit. The patient died of bronchopneumonia after a disease duration of 12 years.

## Neuroimaging Findings

**FIG. 2:**
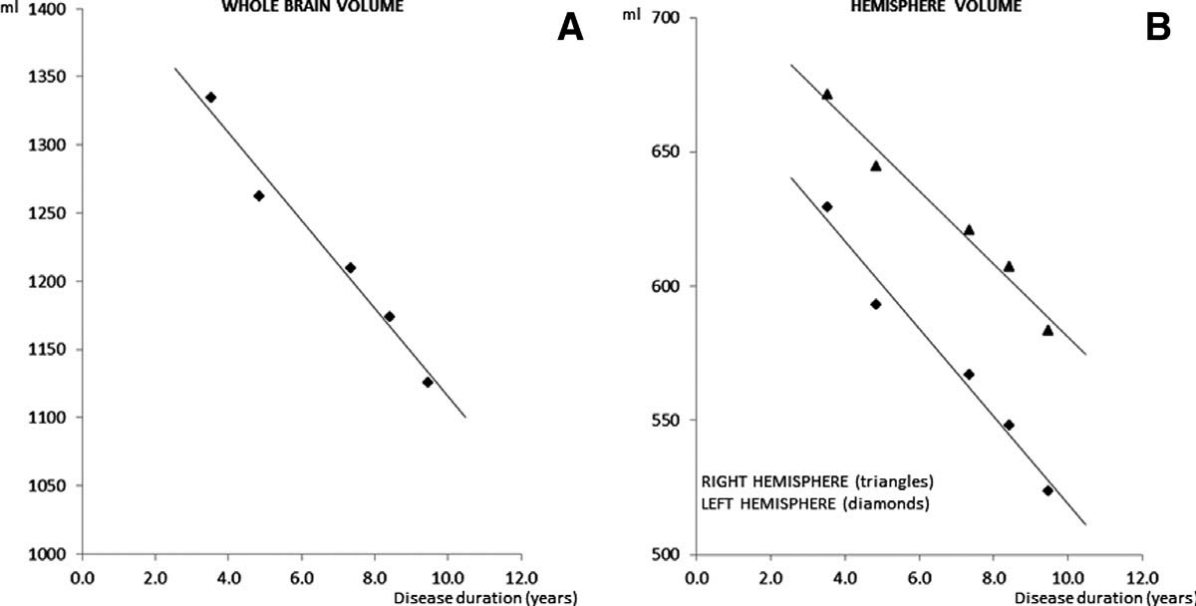
(**A**) Whole brain and (**B**) right/left cerebral hemisphere volumes at 3.5, 4.8, 7.3, 8.4, and 9.5 years from symptom onset.

**FIG. 3:**
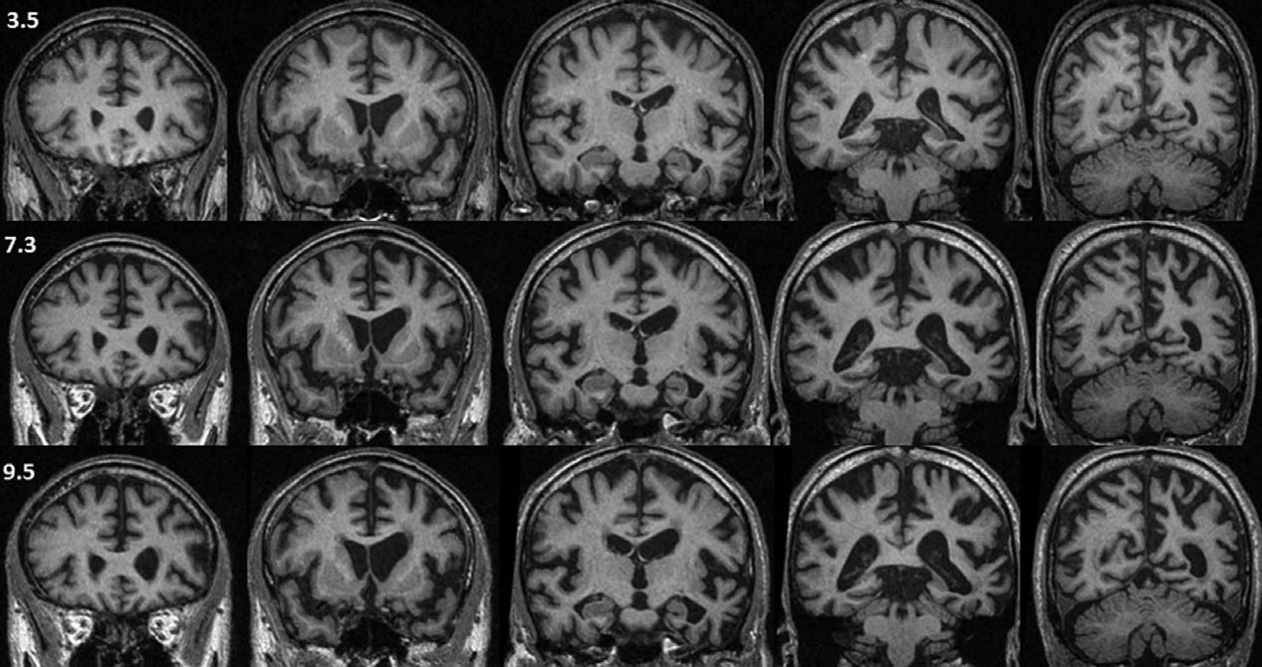
Coronal brain sections from sequential T1-weighted volumetric MRI at 3.5, 7.3, and 9.5 years from symptom onset as indicated. More anterior sections are shown on the left and more posterior sections on the right; the left hemisphere is displayed on the right in all sections.

Volumetric T1 brain MRI was performed at 3.5, 4.8, 7.3, 8.4, and 9.5 years from symptom onset, as part of a longitudinal research study (Figs. 2 and 3). Figure 2 shows the change in brain volume (Fig. 2A) and right and left cerebral hemisphere volumes (Fig. 2B) over time—the rate of whole brain atrophy was 2.6% per year. The left hemisphere was found to be smaller than the right hemisphere when initially measured, with the rate of left hemisphere atrophy (2.8%) greater over time than the rate of right hemisphere atrophy (2.2%). Figure 3 confirms the asymmetry of atrophy, with greater volume loss in the left hemisphere, particularly posteriorly. There was progressive atrophy over time, particularly affecting the left parietal and posterior temporal lobes, but also the frontal lobe and hippocampus, and also increasing involvement of the right hemisphere, particularly posteriorly. However, cerebral atrophy remained asymmetrical, even 9.5 years into the illness.

## Pathological Findings

The brain weighed 1,463 g. The right-half brain (weight, 760 g) was fixed and examined. The frontal lobe was of mildly reduced height, with mild reduction of bulk of the frontal white matter. Coronal slices revealed a normal-sized lateral ventricle and a cortical ribbon of normal width. In the brainstem, the substantia nigra (SN) was markedly pale and the locus coeruleus was indiscernible, but the remaining deep gray structures had a normal appearance.

**FIG. 4:**
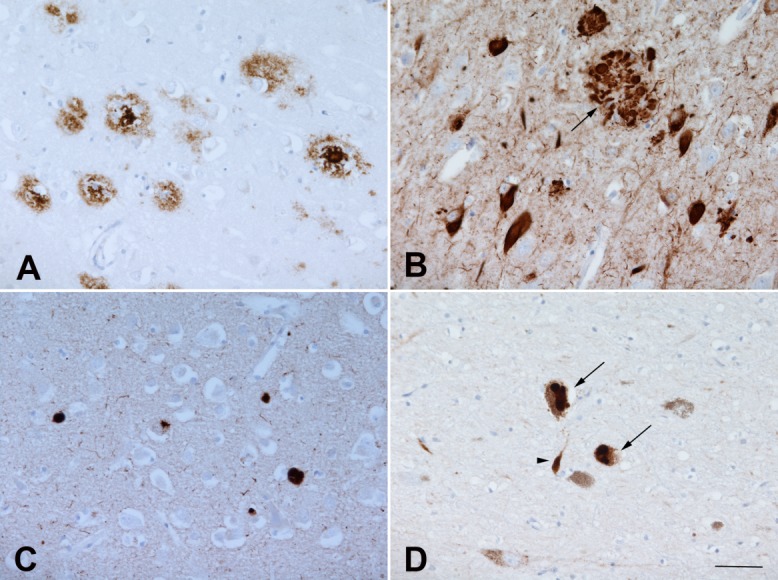
Alzheimer pathology consisting of Aβ immunoreactive diffuse and mature deposits with cores were present in the neocortex (**A,** temporal cortex) in addition to tau pathology in the form of neurofibrillary tangles, abnormal neurites (arrow), and neuropil threads (**B,** CA1 subregion of the hippocampus). In addition, there was diffuse neocortical LB pathology illustrated in the cingulate cortex (**C**). In the brain-stem LBs (arrows) and LNs (arrowhead) were present in the SN, locus coeruleus, and dorsal motor nucleus of the vagus (**D,** SN). (**A**) Aβ IHC; (**B**) tau IHC; (**C** and **D**) α-Syn IHC. Bar in (**D**) represents 50 μm in all panels. [Color figure can be viewed in the online issue, which is available at http://wileyonlinelibrary.com.]

Tissue blocks were selected for histological examination and sections were stained using hematoxylin and eosin supplemented by immunohistochemistry (IHC) in selected regions using the following primary antibodies: Aβ (1:100; Dako, Ely, UK), tau (1:600, AT8 clone; Autogen Bioclear, Wiltshire, UK), TDP-43 (1:800; Abnova, Taipei, Taiwan), and alpha-synuclein (α-Syn) (1:50; Vector, Peterborough, UK). Examination of neocortical regions and the hippocampus revealed frequent diffuse and neuritic Aβ plaques (Fig. 4). Aβ plaques were also found in the striatum and midbrain, but not in the cerebellum corresponding to Thal phase 4^1^. Tau pathology, in the form of neurofibrillary tangles, neuropil threads, and abnormal neurites, corresponded to Braak and Braak stage V. Overall, there was a high level of Alzheimer's pathology (A3, B3, and C3) according to the recently published consensus criteria, confirming a diagnosis of Alzheimer's disease (AD).^2^ A small number of leptomeningeal and cortical vessels showed patchy Aβ deposition. Synuclein IHC confirmed widespread Lewy body (LB) pathology affecting brainstem and neocortical regions, including the parietal cortex. In the brain stem, the SN and locus coeruleus showed moderate neuronal loss with moderate numbers of LBs in both nuclei (Fig. 4). Frequent LBs and Lewy neurites (LNs) were present in the dorsal motor nucleus of the vagus. LB pathology corresponded to Braak stage 6 and was classified as diffuse neocortical LB pathology.^2^,^3^ With this extent of LB pathology and an additional diagnosis of AD current classification suggests that there is an intermediate likelihood that the pathological findings are associated with a dementia with LBs clinical syndrome.^4^ There was no TDP-43 pathology.

The final neuropathological diagnosis was (1) AD, (2) LB disease, neocortical, and (3) mild cerebral amyloid angiopathy.

## Discussion and Learning Points

The term primary progressive aphasia (PPA) refers to a group of neurodegenerative disorders presenting with language dysfunction as the predominant feature.^5^ Three main clinical subtypes are currently described: a semantic variant, also known as semantic dementia, which presents with a fluent aphasia, anomia, and single-word comprehension difficulties; a nonfluent variant, also known as progressive nonfluent aphasia (PNFA), characterized by effortful speech production resulting from one of or both agrammatism and apraxia of speech; and a logopenic variant, or logopenic aphasia (LPA), in which patients have word retrieval impairment with phonological deficits.^5^ Parkinsonism may occur in association with PPA, particularly the PNFA subtype, but when it does, a corticobasal or progressive supranuclar palsy (PSP) syndrome is the most likely clinical presentation, and the underlying pathology is usually a tauopathy.

An idiopathic Parkinson's disease (PD) phenotype is not usually associated with prominent language impairment: When cognition is impaired, executive dysfunction usually predominates initially and posterior cortical dysfunction often supervenes.^6^ However, more-detailed neurolinguistic studies of PD have recently shown that many patients may have underlying grammatical deficits.^7^

Pathologically, PPA is commonly caused by frontotemporal lobar degeneration (FTLD) pathology with abnormal inclusions of either tau (FTLD-tau) or TDP-43 (FTLD-TDP). PNFA is most commonly associated with the FTLD-tau pathologies of Pick's disease, corticobasal degeneration (CBD), and PSP.^8^ However, there is no simple one-to-one correlation, and other pathologies are also noted less frequently, including AD pathology.^9^ In comparison to the other PPA subtypes, LPA is most commonly associated with AD, rather than FTLD pathology, and can be considered as an atypical variant of AD.^5^,^10^

This patient illustrates several important issues in the diagnosis of PPA and related neurodegenerative syndromes. These issues relate to the characterization of his language presentation as well as the interpretation of the associated parkinsonian syndrome in light of the subsequent neuropathological information. The language features here were felt to be most consistent with the PNFA subtype of PPA; however, these feature showed substantial evolution over time. Of note, this patient was first observed in 2002, 2 years before the initial detailed description of the logopenic variant of PPA.^11^ His initial features were those of word retrieval difficulties, and on neuropsychometry testing, language function was relatively intact apart from impaired sentence repetition. These are the features described as occurring early in LPA. However, he later developed a clear motor speech disorder as well as expressive agrammatism and more-widespread linguistic deficits. Neither a motor speech disorder nor agrammatism is described in LPA, although detailed longitudinal information is lacking. The overall evolution of this patient's syndrome would be more consistent with the PNFA/speech apraxia variant of PPA, although an associated dysarthria may well have been related to his parkinsonian disorder, potentially complicating the linguistic assessment. Parkinsonian disorders are also described less commonly in association with LPA than with PNFA.^12^

Our patient's motor phenotype—an asymmetrical parkinsonian syndrome with bradykinesia, rigidity, and rest tremor—was in keeping with PD, and the neuropathological correlate of this was the finding of loss of pigmented neurons in the SN, with LBs in the SN and other brainstem nuclei. However, the associated language dysfunction in this case is unusual in PD. Asymmetrical parkinsonism is also described as part of corticobasal syndrome (CBS), and the additional features of myoclonus and limb apraxia in this case were in keeping with CBS,^13^ but the disease duration was too long for CBD. Myoclonus can be noted in AD, particularly in atypical phenotypes.^14^

There has been considerable recent interest in the use of neuroimaging to define signatures of tissue pathology in focal dementia syndromes.^8^ Brain MRI in this case (Fig. 2) showed progressive asymmetrical perisylvian atrophy consistent with PPA; however, the value of this profile in predicting underlying pathology remains uncertain. Here, the initial findings of accentuated parietal and posterior temporal lobe atrophy would be in keeping with LPA.^5^,^10^,^11^

This case underlines the value of neuropathological correlation in the retrospective interpretation of clinical deficits for diseases in the PPA spectrum. The neuropathological findings suggest that our patient's cognitive decline was primarily attributable to a focal variant of AD,^9^ whereas his motor syndrome was primarily attributable to brainstem LB pathology. Of course, it is likely that the interaction of these two entities produced a clinical picture that amounted to more than simply the sum of the component pathologies: The motor speech phenotype is one case in point, and this interaction may also have been expressed in the underlying dysfunction of neural circuitry, accounting for the similar clinical time frames of eachdisease. In an era of increased interest in clinicopathological signatures that might be used to guide future disease-modifying therapies, this case illustrates a need for caution: Dual pathology is common, and in the case of common diseases, such as AD and PD, atypical variants need consideration.

Unlike our CPC of a patient with PD and subtle autonomic features in which the dual pathology combined both LB disease typical of PD and striatonigral predominant MSA,^15^ the initial clinical features in this case held the clue to the dual-culprit pathologies: This underscores the importance of early diagnosis of neurodegenerative syndromes, when distinctive phenotypic features are less likely to have become obscured by the pathological spread of disease.
